# White's Dentifrices, &c.

**Published:** 1869-05

**Authors:** 


*White's Dentifrices and Washes.*—We have received from Dr. Samuel S. White samples of the Tooth Powders, Washes, Soap and Pastes, etc., he is now preparing for the profession.

Dr. White is making more of a specialty of these articles than heretofore, and the attractive manner in which they are gotten up is an evidence of great taste. No expense has been spared in their manufacture.

The use of the many nostrums of this character at the present time, the majority of which, to say the least of them, are of doubtful utility, has led to the preparation of Dr. White's articles. He certainly deserves credit for his efforts to discourage the sale of injurious dentifrices.

